# LiDAR and Deep Learning-Based Standing Tree Detection for Firebreaks Applications

**DOI:** 10.3390/s22228858

**Published:** 2022-11-16

**Authors:** Zhiyong Liu, Xi Wang, Jiankai Zhu, Pengle Cheng, Ying Huang

**Affiliations:** 1 School of Technology, Beijing Forestry University, Beijing 100083, China; 2 School of Education, University of Nottingham, Nottingham NG7 2RD, UK; 3 Department of Civil, Construction, and Environmental Engineering, North Dakota State University, Fargo, ND 58102, USA

**Keywords:** firebreaks opening, deep learning, LiDAR, object detection

## Abstract

Forest fire prevention is very important for the protection of the ecological environment, which requires effective prevention and timely suppression. The opening of the firebreaks barrier contributes significantly to forest fire prevention. The development of an artificial intelligence algorithm makes it possible for an intelligent belt opener to create the opening of the firebreak barrier. This paper introduces an innovative vision system of an intelligent belt opener to monitor the environment during the creation of the opening of the firebreak barrier. It can provide precise geometric and location information on trees through the combination of LIDAR data and deep learning methods. Four deep learning networks including PointRCNN, PointPillars, SECOND, and PV-RCNN were investigated in this paper, and we train each of the four networks using our stand tree detection dataset which is built on the KITTI point cloud dataset. Among them, the PointRCNN showed the highest detection accuracy followed by PV-RCNN and PV-RCNN. SECOND showed less detection accuracy but can detect the most targets.

## 1. Introduction

### 1.1. Background

Forest firebreaks are open areas between forests and between forests and villages, schools, factories, etc., to prevent the spread of an expanding fire. Traditional firebreaks (all-light firebreaks) are fuel-free strips, i.e., raw earth strips, constructed on the way to the spread of a potential fire to prevent or deter it. Since the 1990s, a new type of biological firebreak has emerged, a dense planting of broad and thick evergreen broadleaf trees or other fire-resistant species in open areas, blocking sunlight and inhibiting the growth of combustible materials on the surface. The latest shade-type firebreaks, which have the advantages of both whole light and biological firebreaks, were developed by USDA Forest Service technicians [1,2,3,4,5,6].

Traditional firebreak openings are generally established using conventional tools such as brush cutters and chainsaws, which not only have safety concerns but most importantly, also have low work efficiency for large amounts of explosive material clearance. In recent years, with the development of automation, practitioners have adopted intelligent equipment that can efficiently perform firebreak opening clean-up work essentially and systematically through manual remote control such as remote-controlled belt openers. However, the existing approaches of opening up firebreaks still require the manual judgment of the location and diameter information of the trees to be removed, which significantly affects the efficiency of mechanized operations. Therefore, it is important to further advance of mechanized firebreak opening operations in the field to detect standing trees in the firebreak region and obtain information on the approximate locations and sizes of trees.

To recognize the standing trees in firebreak openings, object detection [7] has been applied in the field of autonomous driving in the direction of environmental awareness. Researchers used traditional object detection algorithms in early studies to extract image features and detect pedestrians using histogram of oriented gradients (HOG) and achieved a near 100% detection success rate on the MIT pedestrian database [8]. Later, deep learning networks have also been introduced. For instance, a regional convolutional neural network (R-CNN) [9], (TA-CNN) [10]. Other scholars have applied digital image processing techniques and deep learning-based applications to fruit localization and harvesting [11]. Tang used an improved YOLOv4-tiny model to detect oil tea fruits [12].

With the increase in arithmetic power, researchers have attempted to reconstruct the standing trees scene in three dimensions (3D), through the introduction of LiDAR. Researchers have proposed more point cloud-based deep learning networks [13,14,15,16,17,18,19]. For instance, (PiontNet) network [20], and PiontNet++ [21]. Later, Zhou further processed the point cloud data using voxelization methods [22], Wang et al. added convolutional neural networks to point cloud voxelization to improve the detection accuracy [23] and Lang et al. applied PiontPillars to identify people, cars, and cyclists in point cloud data. Cheng investigates generalized LiDAR intensity normalization and its positive impact on geometric and learning-based lane marking detection [24].

### 1.2. Related Work

In the field of forestry, based on the database from UAV remote sensing and machine vision, machine learning networks have been investigated such as YOLOv3 [25], YOLOv4 [26], and Faster-RCNN [27] to identify spruce and count the number of trees [28]. Some scholars have studied the role of UAV LiDAR data in improving the accuracy of tree volume calculations. Based on UAV and ground-based LiDAR data [29,30]. Wang used a non-linear mixed-effects model and a hierarchical Bayesian approach to explore the feasibility of individual tree AGB estimation for long white larch (Larch Henry) in eight plots in three different regions of the Maoershan Forest Farm, Heilongjiang [31]. The tree identification has been moving towards automation, for instance, Hernandez et al. developed a hybrid pixel- and region-based algorithm to segment the images and automatically extract individual trees in the plantation and estimate their heights and crown diameters [32]. Deep learning has been applied to RGB optical images obtained by drones to classify tree species (AlexNet, VGG-16, and ResNet-50) [33]. Tang et al. used the YOLOv5 target detection network and DBSCAN point cloud clustering method to determine the location of litchi at a distance [34].

As the images taken using cameras are heavily influenced by light, they do not give accurate location and 3D information about the trees. On the other hand, LiDAR point cloud data can directly reflect objects’ locations and 3D information and are less affected by the environments. Traditional object segmentation algorithms have been investigated to obtain 3D point clouds to segment and identify trees in forestry based on LiDAR height data [35,36]. A support vector machine (SVM) classifier has been applied to extract single trees based on airborne LiDAR (light detection and ranging) and hyperspectral data [37]. Further research has been conducted using a handheld CCD camera to take sample maps surrounding the central tree for a video-based point cloud for detecting individual tree trunks in the sample map and estimating the diameter at the breast height of each trunk [38].

However, all the above-described methods may not be able to use in real-time and are highly dependent on more accurate point cloud alignment and 3D reconstruction. Traditional methods require specific knowledge and a complex tuning process as they rely on manually designed extractors for feature extraction. Thus, each method is application-specific and may not be robust enough to be used in general conditions. On the other hand, deep learning [39] is primarily data-driven for feature extraction. It can obtain deep, dataset-specific feature representations based on learning from a large number of samples, which is a more efficient and accurate representation of the dataset, with more robust abstract features extracted, better generalization, and can be end-to-end. To date, the deep learning method has not yet been used to identify trees based on point clouds and to obtain 3D information about trees. For the first time, this study uses a point cloud deep learning method combined with LiDAR to detect standing trees and localize tree point cloud data for autonomous machine operation in firebreaks opening. Multiple deep learning networks were analyzed in this paper using the KITTI dataset [40] and showed good detection results compared to detection for pedestrians and cyclists. Accurate tree objects and their location coordinates were obtained, providing tree information and reference for subsequent unmanned equipment to open up firebreaks. This study is the first to use a deep learning approach to process ground-based 3D LiDAR point cloud data for standing tree objection detection. Unlike airborne LiDAR tree identification, which uses the canopy layer as a feature, ground-based LiDAR can obtain more geometric information by directly identifying standing tree trunks in a planar view. Datasets were created based on the KITTI dataset for training and testing. The deep learning network was debugged to adapt to the detection and recognition of standing trees. Specifically, this paper has two main contributions as detailed below:

(1) It proposes a dataset for stumpage detection produced based on the KITTI dataset. It is also demonstrated that LiDAR point cloud data are a valid data source for standing wood detection.
(2) A deep learning approach in combination with LiDAR is developed to achieve standing timber detection, and analyze the detection accuracy and effectiveness of four-point cloud object detection networks, including the PointPillars, PointRCNN, PVRCNN, and SECOND, on the above dataset.

The remainder of the paper is organized as follows. Section 2 presents the details of the LiDAR data combined with deep learning for stumpage detection methods; Section 3 explains the experimental results, Section 3 provides a discussion, and Section 4 concludes the paper and points out some potential future work.

## 2. Materials and Methods

### 2.1. Data Acquisition and Processing

The data for the following study is from the KITTI object detection dataset, including LiDAR point cloud data and camera image data. However, we only used the point cloud data for training and the image data to validate the results in 2D. We relabeled the 2000-point cloud data from the KITTI dataset, boxed and noted the trees in the data, and aligned the LiDAR coordinate system in the label file with the camera coordinate system based on the calibration file. We created a new target species: Tree. The data set and validation set were delineated, with a total of 2000 in the training set and 2157 in the validation set.

### 2.2. Methods

Deep learning has given rise to many applications in scientific research, such as object detection, semantic segmentation, and tracking of moving objects. Object detection is one of the fundamental projects in the field of computer vision. In recent years, convolutional neural network models in the field of two-dimensional images have gradually matured, and deep learning algorithms in the three-dimensional field are progressively emerging. Usually, there are two types of object detection algorithms. One is the two-step algorithm represented by RCNN, the first step is selecting the candidate frame, and the SECOND is selecting the candidate frame for classification or regression. The other is a single-stage algorithm represented by Yolo and SSD [41], which is a straightforward one-step identification and detection of the class and location of the object, completed using only a convolutional neural network. Both algorithms have advantages and disadvantages; two-step algorithms are highly accurate but tend to be slower in recognition, while single-stage algorithms are faster but less accurate. In 3D object detection, researchers often split and reorganize the two algorithms, taking advantage of both to form a new network.

In this study, PiontPillars, SECOND, and PiontRCNN were used to detect standing trees in the KITTI dataset. The most suitable algorithm for standing tree detection was selected by comparing the recognition results.

A summary of the four deep-learning networks is shown in Table 1.

#### 2.2.1. PiontPillars

**Figure 1:**
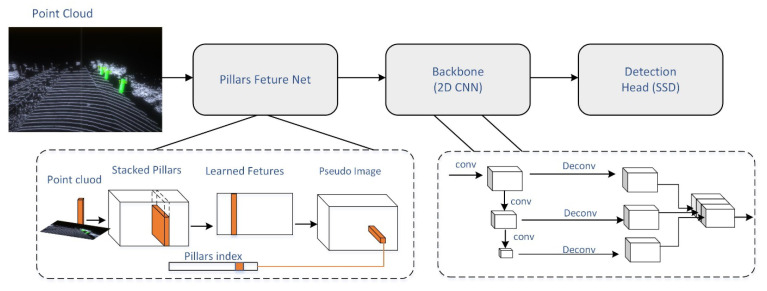
PiontPillars network overview.

PiontPillars [42] takes a different approach to voxelising point clouds, instead transforming the point cloud data into individual point pillars that can be processed as pseudo-images. After inputting the point cloud, the point cloud is divided into a grid, and the point cloud in the same grid is considered a pillar. The net will read the number of pillars, the geometric center of the pillar, and the number of point clouds, with the number of point clouds being the specified value. Suppose the points in the pillar are less than the specified value *n*. In that case, they are filled to *n*, and if more than *n*, random sampling is performed to make the number *n*. This process completes the tensorisation of the point cloud data and then uses a simplified version of the PiontNet to perform data processing and feature extraction on the tensorized point cloud data, and then performs maximum pooling to obtain a pseudo-picture, which is used as the input to the 2D CNN part to extract the picture features further, as shown in the figure. The 3D feature map is upsampled to the same size and then concatenated to fuse the features extracted from multiple convolutions. The network structure is shown in Figure 1.

#### 2.2.2. PiontRCNN

**Figure 2:**
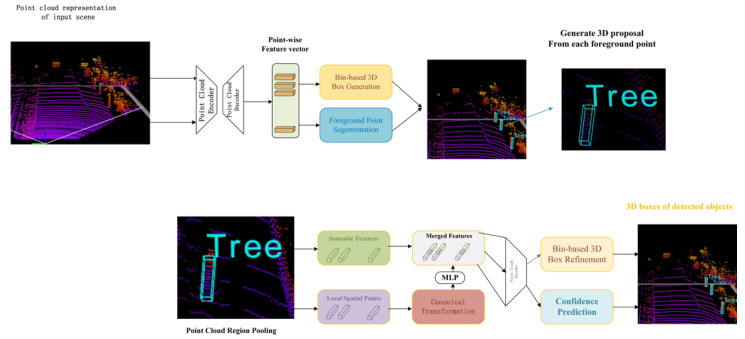
PiontRCNN network overview.

PiontRCNN [43] is used to detect 3D objects directly from the original point cloud and is divided into two main stages. In the first stage, PiontRCNN generates 3D proposals directly from the point cloud in a bottom-up manner, learns point-by-point features, segments the original point cloud, and then generates 3D proposals from the segmented foreground points Based on this bottom-up strategy, a large number of predefined 3D boxes in the 3D space are avoided, and the search space of the generated 3D proposals is significantly limited. The role of the stage 2 network is to combine semantic features and local areas to optimize the proposals in canonical coordinates. Because bounding boxes in 2D object detection can only provide weak supervision for semantic segmentation, the two-stage 3D object detection framework, PiontRCNN, offers high robustness and accurate detection performance. The network structure is shown in Figure 2.

#### 2.2.3. SECOND

**Figure 3:**
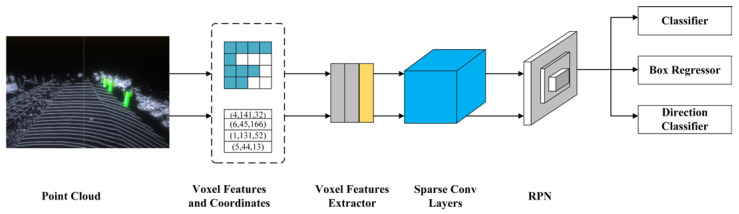
SECOND network overwork.

Yan Y et al. proposed an improved sparse convolutional network and applied it to a LiDAR-based object detection task, which significantly improved the speed of training and inference [44]. In addition, they introduced a new loss function for orientation angle with better performance than other methods. Moreover, a new data augmentation method was introduced for LiDAR-only based learning problems, which improved the convergence speed and performance. The SECOND detection model consists of three components: a voxel grid feature extractor, a sparse convolutional layer (intermediate layer), and an RPN network. The voxel grid feature extractor first groups the point cloud and selects a specific range of points as input to the feature extractor. The original point cloud is then voxel-gridded and the features of each voxel are extracted using the VFE voxel feature extraction network. It would consume a considerable amount of computational resources and time. Here, SECOND uses a submanifold convolution, which limits the sparsity of the output by the sparsity of the input data, thus significantly reducing the computational effort of subsequent convolution operations. An SSD-like network is used as the RPN, consisting of three stages, i.e., (Conv × k + BN + ReLU) × 3, and the output of each stage is then deconvoluted and upsampled to form a feature map. The network structure is shown in Figure 3.

#### 2.2.4. PV-RCNN

Shaoshuai Shi et al. proposed a novel network structure PV-RCNN combining a point_based approach and a voxel_based approach [45].

A two-stage strategy is proposed: in order to reduce the number of voxels needed to encode the scene, the original point cloud is sampled by FPS to obtain a portion of key points to represent the original 3D scene, and then a point-based set abstraction is used to aggregate the key points. Set abstraction module is used to summarize the multi-scale point cloud information by aggregating the voxel features of the points near the key points. In this way, the whole scene can be efficiently encoded into a small set of multiscale keypoint features.

Keypoint-to-grid RoI feature abstraction step: extracting summarized grid point features from keypoints. Considering that each 3D proposal corresponds to the location of its grid points, the proposed RoI-grid pooling module uses a multiple-radius keypoint SA layer for each grid point to aggregate keypoint features with multi-scale contextual information to obtain grid point features. Then, all grid point features in the proposal can be jointly used to optimize the proposal.

The VSA module is used to encode multiscale semantic features from the 3D CNN feature body to the keypoint. Here, the SA operation proposed by PointNet++ is used to aggregate the voxel-level feature volume. That is, the points around the keypoint are now regular voxels with multiscale semantic features, rather than adjacent raw points learned from PointNet to the features. Given a range threshold, the voxels whose distance from the keypoint is less than this threshold are the neighboring voxels of the keypoint; using the PointNet block to obtain the features of the keypoint based on the features of the neighboring voxels; the voxel CNN of PV-RCNN has 4 layers, so each keypoint can obtain 4 features, and different range thresholds can be set in these four layers to aggregate the features of different sensory fields. In these 4 layers, different range thresholds can be set to aggregate the local voxel features of different sensory fields, so that richer feature information can be obtained, and finally, these 4 features are concatenated to generate the multi-scale semantic features of that key point.

For each 3D RoI, RoI-grid pooling is proposed to aggregate the key point features to the RoI grid points with multiple receptive fields. Uniformly sample 6 × 6 × 6 grid points within each 3D proposal; use set abstraction operation to find the nearest neighboring key points within radius r for each grid point and use PointNet to aggregate the features of grid points from the nearest neighboring keypoint features.

**Figure 4:**
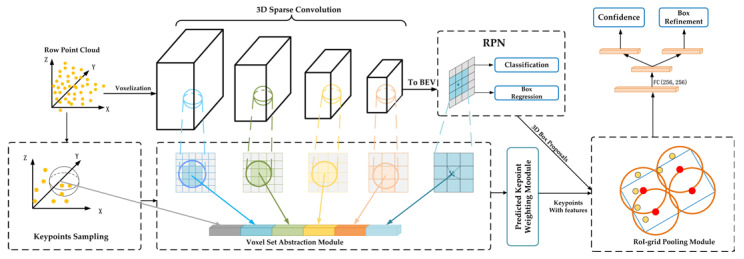
PV-RCNN network structure.

The confidence prediction branch is trained in the proposal and ground-truth boxes using the previous 3D IoU. The network structure is shown in Figure 4.

### 2.3. Training Strategy

We modified the original network in order to apply it to stumpage detection. In order to have a better migration effect, we removed all the original labels and added a completely new class (tree) based on the KITTI dataset, and created a configuration file specifically for this category to set up the configuration of the network.

In order to adapt the size of the model and the object to be detected, we modified the AnchorSize parameter in the configuration files of PointPillars and SECOND. AnchorSize indicates the size of the prediction frame generated by the model for different objects, different detection objects need to set different AnchorSize. Referring to the AnchorSize of Pedestrian in PointPillars [0.6, 1.76, 1.73] and combining with the experiment, we modify the AnchorSize parameter in PointPillars to [0.6, 1.76, 2.73], and set the AnchorSize parameter in SECOND to [0.6, 0.8, 1.73]. In order to improve the accuracy of the prediction, we use a score threshold to keep only those prediction frames with prediction confidence greater than the score. By setting the score threshold, we can filter most of the incorrect prediction frames and keep the correct ones.

In this study, we annotated and produced the KITTI dataset. A total of 3560 tree samples from 2000-point cloud images were included to train three object detection models: PiontPillars, PiontRCNN, and SECOND. They were validated using multiple validation data to identify tree objects detected in the point cloud data and to draw prediction boxes. We use the same dataset for training, with different network structures all identifying standing tree objects but with varying accuracy. In the process of training deep learning networks, the settings of hyperparameters have a great influence on the training results and accuracy, and the settings of network parameters in this study are shown in Table 2.

PointPillars is an improved algorithm for SECOND, so both have the same epoch of 160, but the learning rate adjustment strategies are different. PointRCNN and PV-RCNN are both RCNN-based point cloud detection algorithms, with slightly different epoch settings of 70 and 80, respectively, but both adopt the same learning rate adjustment strategy, increasing the learning rate as the step increases, reaching the maximum learning rate at nearly half of the training time, and then gradually decreasing the learning rate until the learning rate increases. Then, the learning rate is gradually reduced until the learning rate tends to zero.

All four networks are implemented in PyTorch and trained using NVIDIA RTX 5000 GPUs. In contrast to using pre-trained models, the model parameters in our experiments were trained from scratch. To prevent model overfitting, batch normalization and rejection layers are used in each 3D convolutional layer. To reduce the size of high-dimensional point cloud data, averaging pools are used in each 2D convolutional layer.

## 3. Results and Discussion

**Figure 5:**
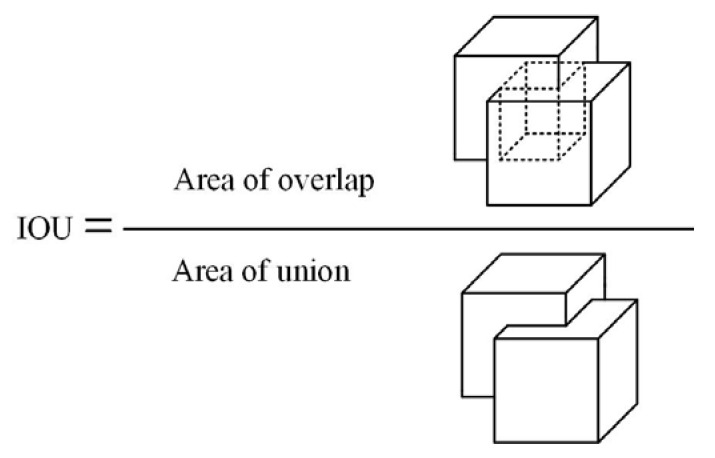
Three-dimensional IOU schematic.

In this study, the accuracy of several networks was obtained by training and validating the neural network. Accuracy (P), recall (R), and average precision (AP) were used in this study. AP is the area under the accuracy and recall curves. It can be used to assess the detection model’s accuracy and analyze the detection effectiveness of individual categories. The model used in this study was a 3D object detection model. Unlike the 2D IOU, which is the intersection and merging ratio of predicted and actual frames, the 3D IOU is the intersection and merging ratio of volumes, as shown in Figure 5.

For a pair of detection boxes (det box) and real boxes (gt box), their IOU value was calculated. If it exceeds the threshold of 0.5, the object is considered to be successfully detected. If the IOU is less than the threshold of 0.5, the detection is deemed unsuccessful. The accuracy rate P is defined in Equation (1) as:

(1) $$P=\frac{TP}{TP+FP}$$

Recall *R* is defined as Equation (2) as:

(2) $$R=\frac{TP}{TP+FN}$$

where *TP* is true positive, indicating that the result is correct, i.e., the detection box and label box are of the same category, and IOU is >0.5. *FP* is false positive for incorrectly identified objects, and *FN* is true negative for undetected objects. AP is calculated by assuming that there are M positive cases in N samples, which leads to M recall values (1/M, 2/M, …). The larger the AP value, the better the model effect.

The KITTI dataset was used as the training set and test set for this study. So, the accuracy results obtained were compared by using the network to recognize two other objects. We relabeled the trees in the dataset and trained them. The final evaluation yielded accuracies close to those of pedestrians and cyclists trained by the network’s original authors. KITTI classified object detection into three modes (easy, moderate, and difficult) by the criteria of degree of occlusion and truncation. As shown in Table 3, in an easy mode, the minimum bounding box height is 40 pixels, the maximum occlusion level is fully visible, and the maximum truncation is 15%. In a moderate level, the minimum bounding box height is 25 pixels, the maximum occlusion level is partially visible, and the maximum truncation is 30%. At a difficult mode, the minimum bounding box height is 25 pixels, maximum occlusion level is difficult to see, and the maximum truncation is 50%.

Through network training and testing, Table 4 shows the average accuracy of the four networks for detecting pedestrians and identifying trees. PiontPillars identified pedestrians with an accuracy of 52.08%, 43.53%, 41.49%, while the detection results of our first produced tree dataset could achieve similar results, 49.24%, 41.32%, 40.54% under the three modes, respectively. PiontRCNN’s recognition accuracy of pedestrians was 49.43%, 41.78%, and 38.63% under the three modes, while the detection for the tree dataset we made showed better results with 53.68%, 52.90%, and 51.05%, respectively. The detection results for the SECOND of pedestrian were 51.07%, 42.56%, 37.29%, but the detection results for the tree dataset were not satisfactory, with 22.80%, 21.96%, and 20.78%, respectively. The detection results for the PV-RCNN of pedestrian were 53.28%, 52.56%, 50.82%, and the detection results for the tree dataset were comparative, with 54.29%, 47.19%, and 43.49%, respectively. It can be seen that PV-RCNN has the best migration learning and the accuracy exceeds the accuracy of pedestrian objection detection.

**Figure 6:**
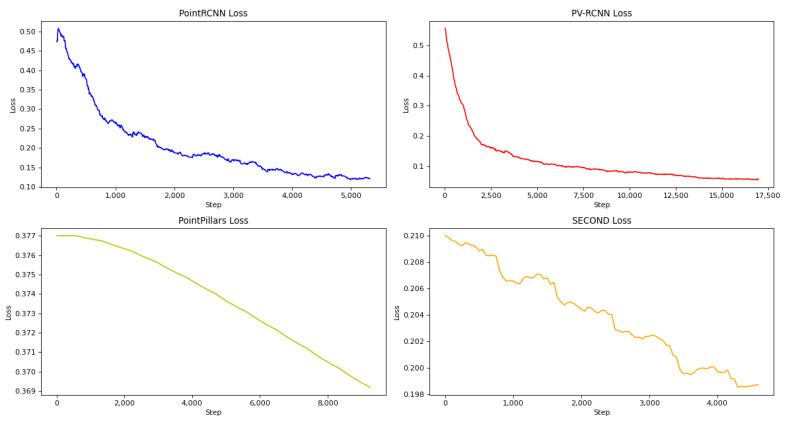
Loss curves of the four networks.

The final loss curves and segmentation results of the four networks are shown in Figure 6.

A common metric to evaluate the speed of an inspection task is the frame per second (FPS), which is the number of images that can be processed in one second. To compare the different algorithm FPS, we used NVIDIA RTX 5000 GPU for testing in order to compare the detection times of the four networks. The detection accuracy of several networks, i.e., mAP, was also compared. The detection times of the four models are shown in Table 5.

**Figure 7:**
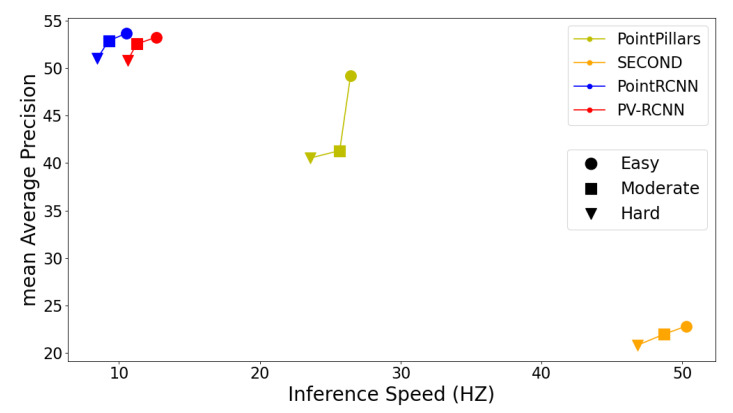
Model detection performance (mAP) versus speed (Hz) in KITTI dataset of our newly created class.

The FPS of each network is calculated from the detection time, and a comprehensive evaluation of the network is performed by combining each performance index. The comparison results of several models are shown in Figure 7.

The horizontal axis is how often the model processes data, i.e., how much data it can process per second, and the vertical axis is the mAP of the model, which indicates the accuracy of the prediction. The circles in the figure indicate the performance of the model on the KITTI EASY level, the squares indicate the performance of the model on the KITTI MODERATE level, and the triangles indicate the performance of the model on the KITTI HARD level, and the same color indicates the same model.

From figure, we can see that PointRCNN and PV-RCNN have higher prediction accuracy but lower data processing speed, SECOND has faster data processing speed but lower prediction accuracy, and PointPillars has more moderate prediction accuracy and data processing speed.

**Figure 8:**
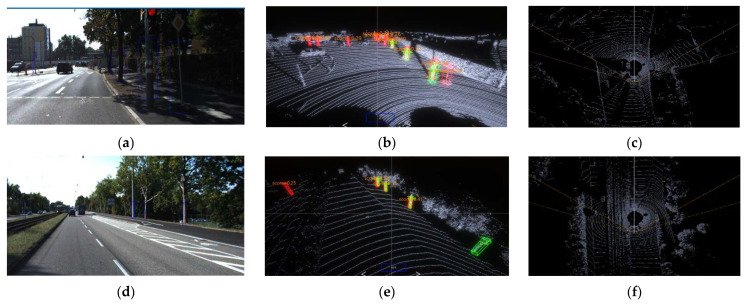
Test results of PointPillars. (**a**) Camera image; (**b**) 3D view of the (**a**); (**c**) a bird’s eye view of (**a**); (**d**) camera image; (**e**) 3d view of the (**d**); (**f**) a bird’s eye view of (**d**).

Figure 8 shows the detection results of the PointPillars network model, showing six high-quality objection detection results. It can be seen in Figure 8b,e that the PointPillars has a good ability to predict densely aligned targets, except for the target bounding box that matches the true value, part of the detection results marked with the true value is not given but the detection results are confirmed to be correct. Figure 8a,d are the 2D camera images of the scene corresponding to Figure 8e,d, respectively. It can be seen that the detected standing wood target is also marked with a blue 2D box. The coordinates of the target are obtained by the coordinate conversion of the detected target in the 3D point cloud after the calibration file of LiDAR and camera, and the corresponding size of the 2D detection box is generated in the corresponding left out. Figure 8c,f are the bird’s eye view of Figure 8b,d, respectively, which can show the detection results in the top view angle. Figure 8b shows that some objects with similar shapes to tree trunks such as utility poles are also detected as standing trees, which is due to the point cloud features of objects such as utility poles being similar to the tree features learned by the network. The distant tree targets are not detected, which may be because the target is too far away, the point cloud is too sparse, or the feature extraction is not sufficient to be recognized as the correct category. Another potential reason may account for that the PointPillars limits the detection distance of targets to 48 m and targets beyond that range cannot be detected.

**Figure 9:**
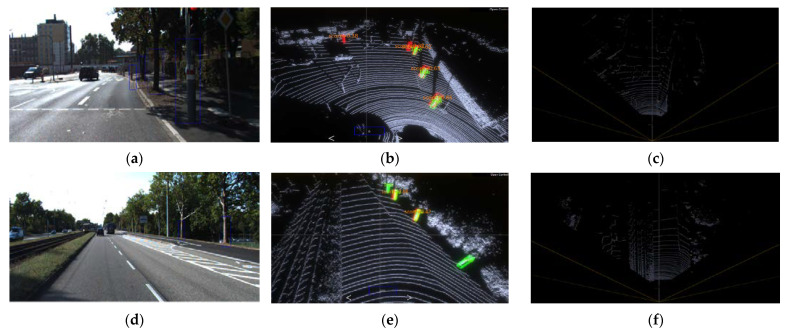
Test results of SECOND. (**a**) Camera image; (**b**) 3D view of the (**a**); (**c**) a bird’s eye view of (**a**); (**d**) camera image; (**e**) 3D view of (**d**); (**f**) a bird’s eye view of (**d**).

Figure 9 shows the detection effect of the SECOND network model, showing six high-quality objection detection results. In Figure 9b, it can be seen that SECOND detects the target outside the bounding box that matches the true value, and some of the detection results marked with the true value are not given, and the detection results are confirmed to be correct, while some of the marked true values in Figure 9e are not detected. Figure 9a,d is the 2D camera images of the scenes corresponding to Figure 9b,e, respectively, and it can be seen that the detected standing wood target is also marked with a blue 2D box, and like PointPillars, the coordinates of this target are also obtained by transforming the coordinates of the detected target in the 3D point cloud through the calibration file of the LiDAR and camera and generating a 2D detection box of the corresponding size on the corresponding left side. Figure 9c,f is the bird’s eye view of Figure 9b,d, respectively, showing the detection results at the top view angle. In Figure 9b, we show that some detection results are correct without the true values because the point cloud features of these misidentified objects are similar to the tree features learned by the network, while the missed detection in d is due to the fact that the true values are marked beyond the detection range limited by the network.

**Figure 10:**
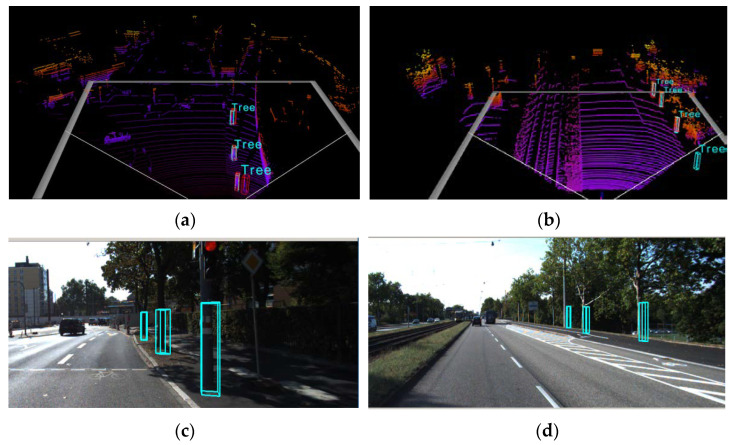
Test results of PointRCNN. (**a**) Three-dimensional view of the (**c**); (**b**) Three-dimensional view of the (**d**); (**c**) camera image; (**d**) camera image.

Figure 10 shows the detection effect of the PointRCNN network model, showing four sheets of high-quality objection detection results. In Figure 10a,b, it can be seen that PointRCNN detects all the target bounding boxes that match the true value, while in Figure 10b, some of the labeled true values are not detected, and the missed detection in Figure 10b is due to the fact that the labeled true values are beyond the detection range marked by the white boxes limited by the network. Figure 10c,d is the 2D camera images of the scenes corresponding to Figure 10a,b, respectively, and it can be seen that the detected standing wood target is also marked by the green-colored 2D boxes. As in the previous two models, the coordinates of the target are also obtained by transforming the coordinates of the detected target in the 3D point cloud through the calibration files of the LiDAR and the camera and generating a 2D detection box of the corresponding size on the corresponding left side.

**Figure 11:**
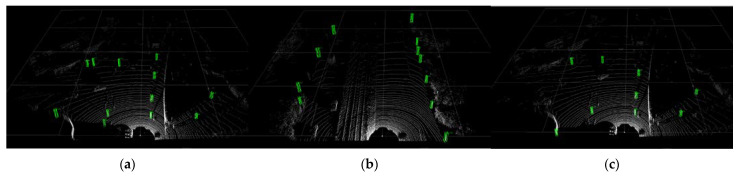

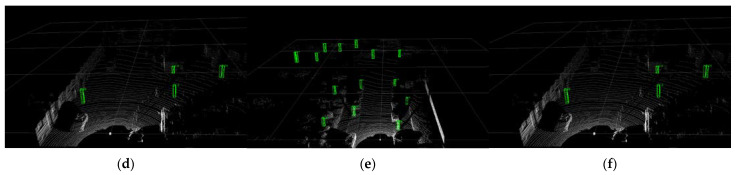
Test results of PV-RCNN. (**a**) Three-dimensional view (**b**) Three-dimensional view (**c**) Three-dimensional view (**d**) Three-dimensional view (**e**) Three-dimensional view (**f**) Three-dimensional view.

Figure 11 shows the detection result graph of the PV-RCNN network model, showing six high-quality objection detection results, Figure 11a–f is different point cloud images, the detection results of this model only mark the detection results, and not marked with the true value, but it is confirmed that the detection results are correct, and there is no missed detection, and the detection effect is good.

## 4. Conclusions

For the first time, this paper combines deep learning with LiDAR data to detect the opposite tree. By training four network models, the most suitable model for detecting standing trees in fire escapes was determined. This method proposed yielded better results than the traditional manual observation solutions, which not only provided better technical support for the research on intelligent devices for opening fire forest roads, but also had important practical significance for opening up intelligent firebreaks.

The experimental results showed that the model could effectively identify trees, but in practice, in order to develop vision systems for intelligent devices that open up firebreaks, the geometric information of standing trees needs to be extracted, such as tree diameter. Future work is devoted to improving the data annotation and network models, so as to further improve the accuracy of detection. Future studies in this area can also explore new vertical tree information detection algorithms, so as to provide more accurate information for the opening equipment.

## Figures and Tables

**Table 1:** Network structure and function overview.

Nets	Layer 1	Layer 2	Layer 3
PointPillars	Feature net Converting point clouds to sparse pseudo-images	Backbone 2D convolutional processing for high-level representation	Detection head Detect and return to 3D frame
SECOND	Features extractor Extraction of voxel characteristics	Sparse convolution Sparse point cloud convolution	RPN Detect and regress target 3D frames
PointRCNN	Point wise feature vector Extraction of voxel characteristics	Former attractions split There are two convolutional layers composed. Classify the points	Generate 3D frames Detect and regress target 3D frames
PV-RCNN	Voxelization Voxelize point cloud data	Sparse convolution 3D convolution of point clouds	RPN Detect and regress target 3D frames

**Table 2:** Hyperparameter setting for four networks.

	Networks	PointPillars	PointRCNN	SECOND	PV-RCNN
Training Parameter					
Epoch		160	70	160	80
Initial learning rate		0.0002	0.0002	0.0003	0.001
Minimum learning rate		0.0002	2.05 × 10^−8^	0.0003	1.00 × 10^−7^
Maximum learning rate		0.0002	0.002	0.00294	0.01
Batch size		2	4	4	2

**Table 3:** KITTI 3D objection detection difficulty grading standard.

Index Level	Difficult	Moderate	Easy
**Pixels height**	>25	>25	>40
**Degree of shading**	Hard to detect	Partially visible	All visible
**Maximum cut-off**	<50%	<30%	<15%

**Table 4:** Comparison of detection results of four network models.

Method	Modality	Tree			Pedestrian		
		Easy	Moderate	Hard	Easy	Moderate	Hard
**PiontPillars**	LiDAR	49.24%	41.32%	40.54%	52.08%	43.53%	41.49%
**PiontRCNN**	LiDAR	53.68%	52.90%	51.05%	49.43%	41.78%	38.63%
**SECOND**	LiDAR	22.80%	21.96%	20.78%	51.07%	42.56%	37.29%
**PV-RCNN**	LiDAR	53.28%	52.56%	50.82%	54.29%	47.19%	43.49%

**Table 5:** Detection time.

Model\Degree	Easy	Moderate	Hard
**PointPillars**	0.038 s	0.039 s	0.042 s
**SECOND**	0.020 s	0.021 s	0.021 s
**PointRCNN**	0.095 s	0.108 s	0.118 s
**PV-RCNN**	0.079	0.089	0.094

## Data Availability

Not applicable.
